# Myasthenia Gravis and Stroke in the Setting of Giant Cell Arteritis

**DOI:** 10.1155/2013/505686

**Published:** 2013-05-28

**Authors:** Elli-Sophia Tripodaki, Sotirios Kakavas, Ioanna Skrapari, Dimitrios Michas, Giorgios Katsikas, Charikleia Kouvidou, Theodoros Gounaris, Euaggelia Sioula

**Affiliations:** ^1^1st Department of Internal Medicine, Evangelismos General Hospital, Ypsilanti 45-47, 10676 Athens, Greece; ^2^9 Kosma Aitolou, Herakleio, 14121 Athens, Greece; ^3^Department of Neurology, Evangelismos General Hospital, Ypsilanti 45-47, 10676 Athens, Greece; ^4^Department of Rheumatology, Evangelismos General Hospital, Ypsilanti 45-47, 10676 Athens, Greece; ^5^Department of Pathology, Evangelismos General Hospital, Ypsilanti 45-47, 10676 Athens, Greece

## Abstract

This case report concerns the diagnosis of two independent chronic diseases in a patient hospitalized for stroke, myasthenia gravis (MG) and giant cell arteritis (GCA). MG has been found to be associated with several diseases, but there are very few cases documenting its coexistence with GCA. We report the case of a 79-year-old woman initially hospitalized for stroke. Patient's concurrent symptoms of blepharoptosis, dysphagia, and proximal muscle weakness were strongly suggestive of myasthenia gravis. The persistent low-grade fever and elevated inflammatory markers in combination with the visual deterioration that developed also raised the suspicion of GCA. Histological examination confirmed GCA, while muscle acetylcholine receptor antibodies were also present. Even though in medicine one strives to interpret a patient's symptoms with one diagnosis, when one entity cannot fully interpret the clinical and laboratory findings, clinicians must consider the possibility of a second coexisting illness.

## 1. Introduction

Myasthenia gravis (MG) is an immune-mediated disease that compromises the postsynaptic membrane of the neuromuscular junction and usually leads to symptoms of fatigability and decreased muscle strength. It is characterized by the production of antibodies to the *n*-acetylcholine receptor (AChR) as a dominating feature. Some MG patients, who do not have AChR antibodies, have antibodies to muscle-specific kinase (MuSK) or other muscle proteins such as titin, ryanodine receptor (RyR), and voltage-gated potassium receptor. MG remains a challenging medical diagnosis due to its fluctuating symptoms and similarity to other disorders. A clinical diagnosis (patient history and findings of fluctuating and fatigable weakness, particularly involving extraocular and bulbar muscles) may be confirmed by laboratory testing including (1) pharmacologic testing with edrophonium chloride that elicits unequivocal improvement in strength; (2) electrophysiologic testing with repetitive nerve stimulation (RNS) studies and/or single-fiber electromyography (SFEMG) that demonstrates a primary postsynaptic neuromuscular junctional disorder; (3) by serological demonstration of AChR or MuSK antibodies [1].

Giant cell arteritis (GCA) also referred to as cranial arteritis or temporal arteritis is a vasculitis of predominantly large- and medium-sized arteries and is the most prevalent of the systemic vasculitis syndromes. Inflammation of the vessel wall is characterized by infiltration of T cells and macrophages, presence of eponymous giant cells, granulomatous lesions, intimal hyperplasia, and destruction of elastic fibres [2]. Symptomatic vessel inflammation usually involves cranial branches of arteries originating from the aortic arch, including the superficial temporal artery, the ophthalmic artery, and the posterior ciliary arteries [3]. The incidence of GCA increases after the age of 50 and peaks between 70 and 80 years of age, with a current prevalence of approximately 20/100000 in Central Europe. Women are affected more frequently than men (3 : 1). The disease is associated with polymyalgia rheumatica. Typical symptoms owing to temporal artery involvement in GCA include unilateral headache, jaw claudication, and visual loss due to ischaemic optic neuropathy. When arteries, however, other than the temporal artery are involved (in more than half of GCA cases), symptoms are often atypical and may involve arm claudication, signs of regional or global cerebral hypoperfusion, low grade fever, and general malaise. Diagnosis is based on typical clinical symptoms, raised serologic inflammatory markers, response to glucocorticoids, and exclusion of other diseases. The gold standard for diagnosis remains temporal artery biopsy.

MG has been found to be associated with a large number of autoimmune diseases (primary sclerosing cholangitis, autoimmune thyroiditis, systemic lupus erythematosus, rheumatoid arthritis, ankylosing spondylitis, Sjogrens) but there are very few cases documenting the coexistence of MG and GCA.

## 2. Case Presentation

A 79-year-old woman presented to the emergency department with left arm and leg weakness and dysarthria. She had previously experienced several weeks of generalized weakness, malaise, temporal headache, and left palpebral ptosis, without a recording of her temperature. She did not mention any episodes of jaw claudication or scalp tenderness. Her medical history was significant for arterial hypertension, cholecystectomy, and a cataract operation in the right eye. 

On examination, reduced power was noted in her left arm and leg with normal speech and no facial droop. The left palpebral ptosis was noted which upon repeat questioning she insisted to be contained to the left eye lid and did not fluctuate during the day. A low-grade fever was recorded. Physical examination of both temporal arteries showed no abnormalities. Auscultation of the chest revealed a systolic murmur.

Computed tomography (CT) of brain performed upon admission revealed ischaemic leukoencephalopathy but no infarcts, whereas the repeat CT showed subacute infarcts in the right posterior parietal and occipital lobes. Ultrasound of carotids showed no significant stenosis, and ECG revealed a sinus rhythm.

Initial investigations revealed anemia (normochromatic and normocytic) and moderately elevated transaminases. Our patient's erythrocyte sedimentation rate (ESR) was 112 mm/h, and her C-reactive protein level (CRP) was 14.4 (normal < 0.5) mg/dL. 

A lumbar puncture was performed which revealed an absence of cells with normal protein and glucose (the fluid culture that ensued was negative).

A transthoracic cardiac ultrasound showed a mild hypertrophy of the left ventrical, an exertion fraction of 60%, and mild mitral valve regurgitation. Vegetations were not observed. 

A computed tomography of the thorax and upper and lower abdomen was performed which did not reveal any significant findings.

Thyroid function tests were within normal range. Antinuclear antibody assay was negative, as were antineutrophil cytoplasmic antibody, rheumatoid factor, PANCA, CANCA, and anti-Jo1. Complement factors C3 and C4 were elevated. Repeated blood and urine cultures were negative. The Mantoux screening test was negative.

A gastroscopy and colonoscopy were performed which were normal.

Due to the low-grade fever which persisted and the elevated inflammatory markers, despite the absence of other signs or symptoms of infection, broad spectrum antibiotics were initiated, as was antiplatelet therapy (clopidogrel). The low-grade fever and inflammatory markers persisted despite the antibiotic regime.

On the 10th day of hospitalization and although patient's left arm and leg weaknesses were improving, the unilateral blepharoptosis evolved into bilateral blepharoptosis, and she presented with acute onset dysphagia involving liquids and solids, dysarthria, and proximal muscle weakness. She also mentioned deterioration in her vision, which was objectively confirmed with reduced optical acuity and a limitation in the visual fields (completely in the right eye). 

Magnetic resonance imaging (MRI) of brain confirmed the CT findings without revealing significant additional pathologic features. 

Due to the reduced optical acuity and the limitation in the visual fields—although the full clinical picture of the patient could not be interpreted by a probable diagnosis of giant cell arteritis—a temporal artery biopsy as well as skin, muscle, and vessel biopsy was performed. Pending biopsy results, we found it prudent to treat the patient with high-dose intravenous methylprednisolone (1 mg/kg) without delay due to the vision deterioration. We also proceeded to an investigation of serum antibodies to muscle acetylcholine receptors (AChR-Ab) as patient's symptoms of blepharoptosis, dysphagia, and proximal muscle weakness were strongly suggestive of myasthenia gravis (MG). 

**Figure 1:**
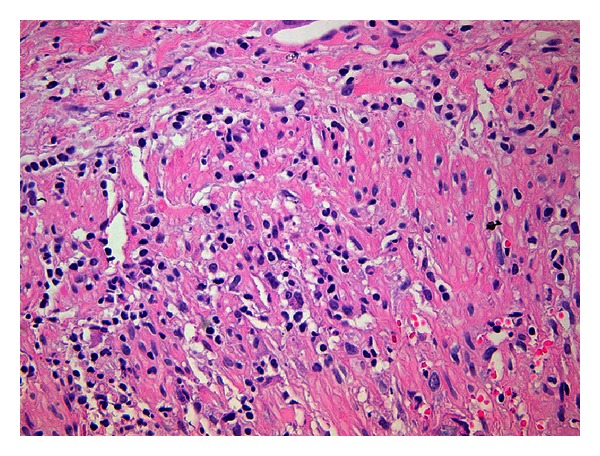
Destruction of the wall by an inflammatory infiltrate containing multinucleated giant cells associated with the internal elastic lamina of the artery (Hematoxylin-Eosin ×40).

Histological examination indeed confirmed giant cell arteritis, while the AChR-Ab were also positive (Figure 1). The skin muscle vessel biopsy did not reveal any pathology.

 The dysphagia, dysarthria, and blepharoptosis showed immediate response to the steroid treatment. Patient's temperature readings returned to normal, and the inflammatory markers (ESR and CRP) gradually decreased, until they returned within normal range.

Upon the result of AChR-Ab, oral pyridostigmine was added to the patient's treatment (60 mg three times daily).

Patient was discharged with high-dose oral steroids (prednisolone 55 mg daily), oral pyridostigmine (60 mg three times daily), and antiplatelet therapy. 

Upon reexamination in two weeks time, due to a slight deterioration in patient's status (blepharoptosis, dysphagia and dysphagia were exasperated), the pyridostigmine dose was increased (60 mg 4 times daily).

Four weeks after she was discharged from our department, the patient presented to the emergency room with dyspnea and palpitations. Upon physical examination, except for tachypnea and tachycardia, an edema of her right leg was noted. ECG showed a sinus tachycardia. She had respiratory alkalosis and elevated D-dimers levels. She underwent a triplex ultrasound which revealed deep vein thrombosis of the right leg, while C/T angiography demonstrated a massive pulmonary embolism. Deep vein thrombosis was probably the result of the limited mobility of the patient due to myasthenia, as the evaluation for thrombophilia was unrevealing.

We continue to reassess the patient monthly in cooperation with our hospital's rheumatologists and neurologists. After the initial two-month-high dose steroid regimen, they will gradually be tapered, depending on the patient's condition.

## 3. Discussion

This case presents clinical interest because it involves the diagnosis of two independent chronic diseases in a patient hospitalized for stroke. Several clinical observations support the idea that a general derangement of the immune system regulation plays an important role in the pathophysiology of MG. MG has been reported to be associated with a number of autoimmune disorders including more frequently rheumatoid arthritis [4], Graves' disease [4, 5], and systemic lupus erythematosus [6] but also Type I diabetes [5]. Moreover, similar genetic associations, overlapping with other autoimmune conditions, including the HLA-B8, DR3 haplotypes and the R620W variants of PTPN22 have been demonstrated in the case of MG [7]. Finally, patients with MG demonstrate a therapeutic response to various immunomodulating therapies, immunosuppressants, and thymectomy [8]. 

These observations suggest that common mechanisms may exist which predispose MG patients for additional autoimmune disorders. In our patient, MG was found to overlap with GCA which to our knowledge is extremely rare. An extensive review of the literature revealed very few cases of the coexistence of MG and vasculitis. In a case series of 25 patients with GCA, only one also had MG [9]. In a more recently published study by Liozon et al. [10], only one case of MG is reported in a series of 250 patients with GCA during a study period of 27 years. We also found one case report describing the concurrence of MG with polyarteritis nodosa [11], as well as a case of myasthenia gravis in the setting of microscopic polyangiitis [12].

In our patient, as was presented in the brief history, there was a time coincidence of the clinical manifestation of the two diseases. The persistent fever, headache, and especially the decrease in optical acuity demanded that the diagnostic followup includes GCA. Nevertheless, solely this diagnosis was not adequate to interpret the entire clinical picture. 

The blepharoptosis was unilateral in the beginning, which is quite unusual in MG [13]. This is why the suspicion of MG intensified when during her hospitalization bilateral blepharoptosis, dysphagia, and proximal muscle weakness were added to the clinical picture. 

It is usual in medicine to strive to interpret a patient's symptoms with one diagnosis. In some cases though when patient's clinical and laboratory findings cannot all be comprehended by one entity, it is imperative that the diagnostic followup investigates the possibility of a second coexisting illness. 

It is worth noting that our patient presented to the ER and was initially hospitalized due to symptoms of left pyramidal syndrome which were found to be caused by an ischemic brain infarct. We speculate that the stress of the stroke led to an exasperation of the symptoms of MG in our patient. We consider the stroke to be associated with the history of arterial hypertension. It is important to add that there are very few cases of concomitant stroke and GCA in the literature. This can be explained by the fact that the inflammatory response in GCA is directed towards the elastic fibres in the media and adventia, which are sharply reduced within about 5 mm of the artery entering the dura. GCA is reportedly the cause of first-ever stroke in only 0.11% of patients. 50%–75% of strokes associated with GCA occur in the vertebrobasilar circulation (compared with 15%–20% of strokes in patients without GCA) [14]. Stroke associated with extracranial involvement in GCA occurs in 3 to 7% of GCA patients, which was not the case in our patient as carotid artery and vertebrobasilar circulation triplex revealed no abnormal findings [15].

## 4. Conclusion 

Myasthenia gravis has been associated with a number of autoimmune disorders; we report a rare case of its coexistence with giant cell arteritis. The diagnosis of GCA can prove to be very challenging especially in the case of the coexistence of a second newly diagnosed disease such as MG. Moreover, clinicians should keep in mind that MG may occur in the setting of other autoimmune diseases. It is of special significance to note how vigilant clinicians must be in rapidly considering the possibility of MG but mainly GCA, especially when it presents with visual disturbances. The early recognition of symptoms and rapid initiation of treatment are critical not only in relieving the patient's symptoms but also in preventing the potentially catastrophic progression of the illness.
